# Remote health workforce turnover and retention: what are the policy and practice priorities?

**DOI:** 10.1186/s12960-019-0432-y

**Published:** 2019-12-16

**Authors:** John Wakerman, John Humphreys, Deborah Russell, Steven Guthridge, Lisa Bourke, Terry Dunbar, Yuejen Zhao, Mark Ramjan, Lorna Murakami-Gold, Michael P. Jones

**Affiliations:** 1 Menzies School of Health Research, Centre for Remote Health, CNR Simpson and Skinner Streets, Postal: PO Box 4066, Alice Springs, NT 0871 Australia; 2 Monash University School of Rural Health, PO Box 91, Strathdale, VIC 3550 Australia; 3 Centre for Child Development and Education, Menzies School of Health Research, Building Red 9, Charles Darwin University, Casuarina campus, Ellengowan Drive, Postal: PO Box 41096, Casuarina, NT 0811 Australia; 4 University Department of Rural Health, The University of Melbourne, PO Box 6500, Shepparton, VIC 3632 Australia; 5 Indigenous Social and Wellbeing Centre, School of Population Health Research, Australian National University, Canberra, Australia; 6 Northern Territory Department of Health, 87 Mitchell Street, Darwin, NT 0800 Australia; 7 Top End Health Service, Northern Territory Government, GPO Box 40596, Area 2C Casuarina Plaza, Casuarina, NT 0810 Australia; 8 Poche Centre for Indigenous Health & Well-Being, Flinders NT, Rubuntja Building – Alice Springs Hospital, PO Box 2234, Alice Springs, NT 0871 Australia; 9 Psychology Department, Macquarie University, North Ryde, NSW 2109 Australia

## Abstract

**Background:**

Residents of remote communities in Australia and other geographically large countries have comparatively poorer access to high-quality primary health care. To inform ongoing policy development and practice in relation to remote area health service delivery, particularly in remote Indigenous communities, this review synthesizes the key findings of (1) a comprehensive study of workforce turnover and retention in remote Northern Territory (NT) of Australia and (2) a narrative review of relevant international literature on remote and rural health workforce retention strategies. This synthesis provides a valuable summary of the current state of international knowledge about improving remote health workforce retention.

**Main text:**

Annual turnover rates of NT remote area nurses (148%) and Aboriginal health practitioners (80%) are very high and 12-month stability rates low (48% and 76%, respectively). In remote NT, use of agency nurses has increased substantially. Primary care costs are high and proportional to staff turnover and remoteness. Effectiveness of care decreases with higher turnover and use of short-term staff, such that higher staff turnover is always less cost-effective. If staff turnover in remote clinics were halved, the potential savings would be approximately A$32 million per annum. Staff turnover and retention were affected by management style and effectiveness, and employment of Indigenous staff.

Review of the international literature reveals three broad themes: *Targeted enrolment into training and appropriate education* designed to produce a competent, accessible, acceptable and ‘fit-for-purpose’ workforce; addressing broader health system issues that ensure *a safe and supportive work environment*; and providing *ongoing individual and family support*.

Key educational initiatives include prioritising remote origin and Indigenous students for university entry; maximising training in remote areas; contextualising curricula; providing financial, pedagogical and pastoral support; and ensuring clear, supported career pathways and continuing professional development.

Health system initiatives include ensuring adequate funding; providing adequate infrastructure including fit-for-purpose clinics, housing, transport and information technology; offering flexible employment arrangements whilst ensuring a good ‘fit’ between individual staff and the community (especially with regard to cultural skills); optimising co-ordination and management of services that empower staff and create positive practice environments; and prioritising community participation and employment of locals.

Individual and family supports include offering tailored financial incentives, psychological support and ‘time out’.

**Conclusion:**

Optimal remote health workforce stability and preventing excessive ‘avoidable’ turnover mandates alignment of government and health authority policies with both health service requirements and individual health professional and community needs. Supportive underpinning policies include:

- Strong intersectoral collaboration between the health and education sectors to ensure a fit-for-purpose workforce;
- A funding policy which mandates the development and implementation of an equitable, needs-based formula for funding remote health services;
- Policies that facilitate transition to community control, prioritise Indigenous training and employment, and mandate a culturally safe work context; and
- An employment policy which provides flexibility of employment conditions in order to be able to offer individually customised retention packages

There is considerable extant evidence from around the world about effective retention strategies that contribute to slowing excessive remote health workforce turnover, resulting in significant cost savings and improved continuity of care. The immediate problem comprises an ‘implementation gap’ in translating empirical research evidence into actions designed to resolve existing problems. If we wish to ameliorate the very high turnover of staff in remote areas, in order to provide an equitable service to populations with arguably the highest health needs, we need political and executive commitment to get the policy settings right and ensure the coordinated implementation of multiple strategies, including better linking existing strategies and ‘filling the gaps’ where necessary.

## Background

Access to high-quality comprehensive primary health care (PHC) services is vital to population health, because these are the most efficient and equitable way to deliver improved health outcomes [1, 2]. This need is not met for residents of remote and isolated rural communities in Australia and other geographically large countries; this is especially the case for remote-living Indigenous people who have unacceptably high levels of morbidity, mortality and poverty, limited access to PHC and high hospitalisation rates [3, 4]. The Remote Health context is also characterised by limited economic activities, socio-economic disadvantage and relatively limited political power [5].

Health care access issues are largely associated with the persistent problem of health workforce under-supply and maldistribution [6]. In remote areas of Australia, the primary care workforce consists mainly of community-based remote area nurses (RANs) and Aboriginal health practitioners (AHPs), supported by visiting medical and allied health staff. RANs and AHPs live and work in small, isolated communities scattered across a vast landscape. The population density of the Northern Territory (NT) of Australia, for example, is 0.2 persons per square kilometre.

Over recent years, policies have increasingly promoted the use of short-term and agency staffing in remote communities [7]. Anecdotally, this has resulted in several undesirable effects, including concern about cost and quality of care, particularly related to continuity of care in a complex, cross-cultural environment [8, 9]. In addition, some studies have suggested that high numbers of short-term nurses contribute to stress encountered by RANs [10]. Similar workforce issues are evident globally [11–13]. Until recently, however, there has been little empirical examination of the use and impact of short-term staff in Australia [14, 15].

We undertook a comprehensive study of workforce turnover and retention in remote communities in the Northern Territory (NT) [16]. The study analysed data from NT Government administrative datasets including hospital admissions, primary health care visits, personnel information, patient travel, government payroll and accounting systems. Primary qualitative data from patients and health service staff were also collected and analysed. In order to inform ongoing policy development and practice in relation to remote area health service delivery, particularly in remote Indigenous communities, we also undertook a narrative review of the relevant international literature to derive the best available empirical evidence relating to remote health workforce retention. In this paper, we offer a synthesis of this literature and the findings of our original research to provide a valuable summary of the current state of international knowledge about improving remote workforce retention.

## Health workforce turnover and retention in remote Australia

Our research revealed an extremely high turnover of resident staff in the 53 NT Government (NTG) remote clinics examined over the period 2013–2015 [17]. Primary turnover of RANs at a clinic level was 148% per annum (p.a.), and for AHPs was less, but still high at 80%. Stability rates were low, but substantially higher for AHPs (76%) than nurses (48%). Only 20% of nurses and AHPs remained working at the same remote clinic 12 months after commencing (RANs 19%, AHPs 27%); half left within 4 months.

**Fig. 1:**
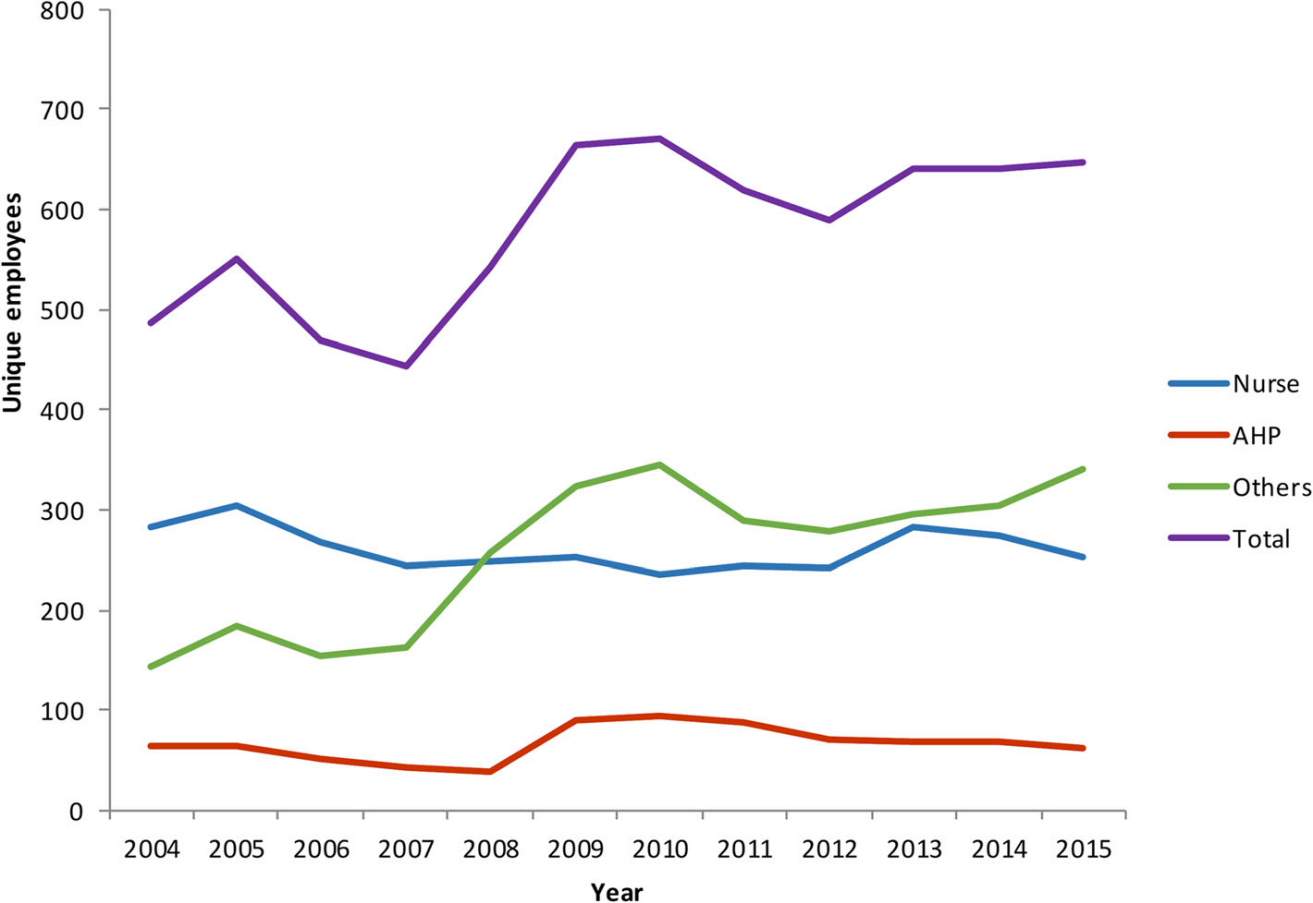
Total number of unique employees by employment category and time, 2004–2015, Northern Territory

**Fig. 2:**
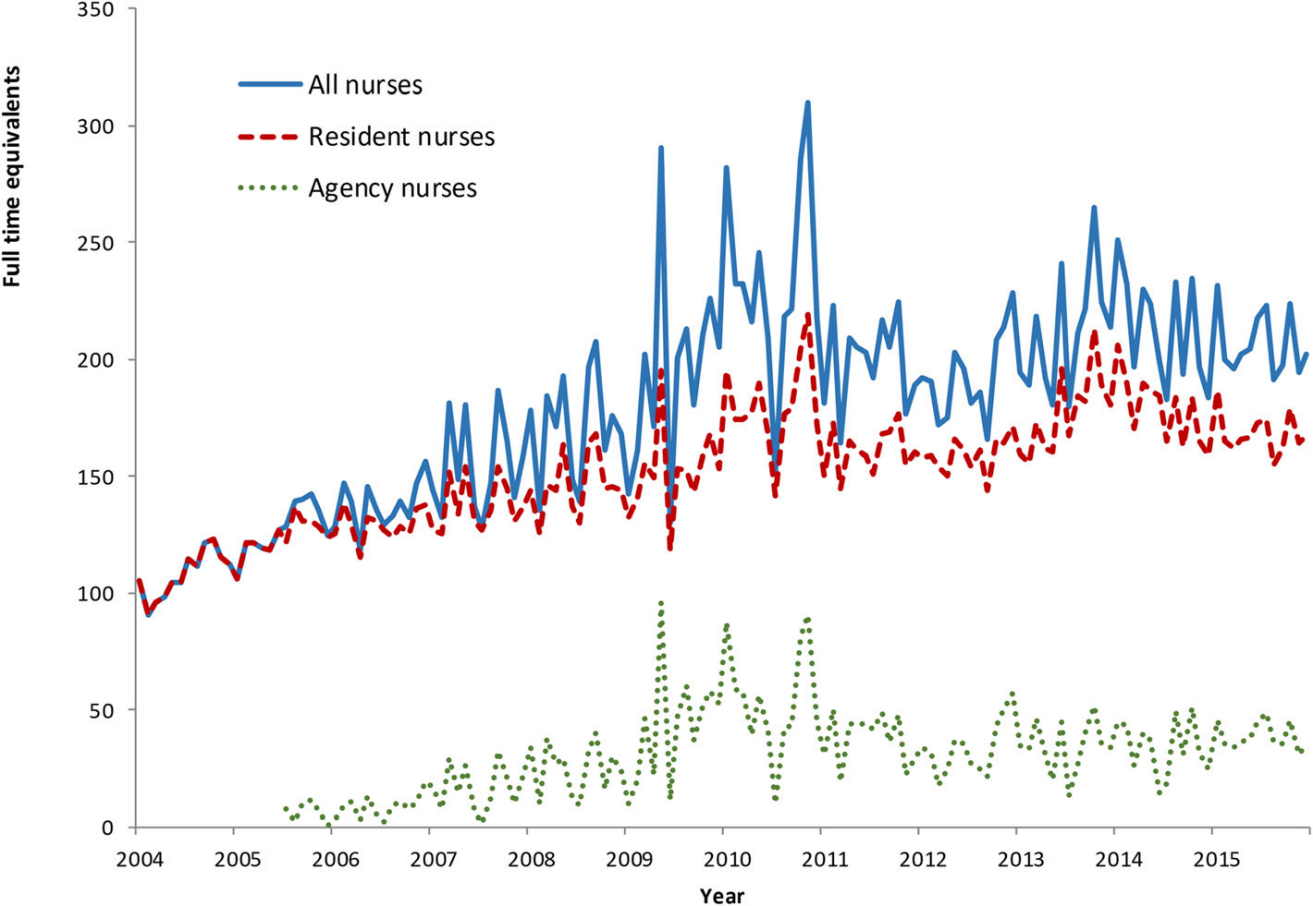
Trends in full-time equivalent agency and NT DOH-employed nurses, 2004–2015, Northern Territory

Longer-term data for the period 2004–2015 showed there were overall increases in workforce supply, especially for administrative and physical grades (labelled ‘Others’ in Fig. 1) [18]. The supply of nurses and AHPs increased from an average 2.6 to 3.2 full-time equivalents (FTE) per clinic, although this varied across clinics. Supply of nurses increased as a result of increased funding associated with the Australian Government Intervention (AGI) in 2007 [19], but this was not a statistically significant rise and subsequent fading of supply was evident. The supply of AHPs also declined after 2010. Agency nursing FTE as a proportion of the total increased over this decade, notably in the post-AGI period (Fig. 2).

The costs of maintaining agency and short-term staffing were high [20]. Notably, the cost differentials between clinics were proportional to staff turnover and remoteness. A 10% higher annual turnover rate generated a A$6.12 increase in costs per consultation. Reducing the turnover of AHPs and RANs from 128 to 40% would have resulted in savings of about A$50 per consultation, equating to a total of A$21 million in savings annually for the NTG.

There was a decrease in the effectiveness of services (as measured by outcomes of hospitalisations and years of life lost) associated with higher use of short-term remote staff [21]. Higher turnover was associated with significantly higher hospitalisation rates and higher average health costs. In contrast, lower turnover was always more cost-effective. Average costs were also significantly higher when higher proportions of agency-employed nurses were employed, with lower use of agency-employed nurses having an 85% likelihood of being more cost-effective. We estimated that if staff turnover in remote NTG clinics were halved, the potential savings to the NTG health system in PHC, travel and hospital costs would be approximately A$32 million p.a., which amounts to 29% of the total NTG remote clinic expenditure in 2015 [20].

Fieldwork in seven remote communities underscored the fragility of workforce status over time. All clinics struggled to completely fill RAN and AHP vacancies. Community members wanted AHPs and other local residents to be employed by the clinic long term; they highly valued the employment of RANs who were both clinically and culturally competent; and wanted on-country nurse and AHP training [22]. Community members also emphasised the importance of building relationships and engaging with local community members for effective PHC. Clinics with higher staff turnover struggled to elucidate and address community needs. They were more focused on immediate and emergency clinical care, and the need to ‘tick off’ performance indicators. Re-visiting these communities 1–2 years after the initial fieldwork found very few of the staff interviewed still working in the same communities. Many RANs identified the delivery of an effective PHC service as a crucial issue, usually in its perceived absence.

## What is the international evidence relating to improving remote health workforce retention?

In order to contextualise our findings and the resultant policy implications, we undertook a narrative review of the international literature. We drew on close to a century of combined remote and rural workforce research experience and knowledge of the authors to identify key review papers, and used a snowballing approach therefrom. We drew on English language publications since 2010 (the year that two of the authors published a systematic review on this subject), with direct relevance to remote health workforce in high-income countries [23]. We found that whilst health staff in small, remote communities usually turn over at a higher rate than in rural or urban settings [24], the extremely high turnover of resident staff encountered in our research was considerably greater than other studies of hospital nursing turnover in Australia (15.1%), New Zealand (44.3%), the United States of America (26.8%) and Canada with 19.9% [11]^;^ and NT RANs (57%) [25]. Agency nurses made up 15% of nurses employed, but this was likely an underestimate as we were unable to quantify those agency nurses directly employed by NT Department of Health (DOH). NT DOH estimated the proportion as closer to 50% [14].

In light of this increased reliance on short-term staffing, very high avoidable turnover, the negative impact on effectiveness and cost, and other local and international evidence linking high turnover to poorer continuity of care [8, 26], there is clearly an urgent need to stabilise the remote workforce to ensure effective, efficient and culturally safe PHC services. Accordingly, we investigated the current state of knowledge about improving retention and how this evidence could be applied to the remote health workforce.

Whilst retention strategies need to be sensitive to local and national context, thematic analysis by the team of conceptual models and evidence in the literature (including reviews of workforce retention strategies) and subsequent synthesis through iterative discussions resulted in several consistent themes emerging from literature covering different geographical areas and dealing with different health professional groups (for example [12, 23, 27],). Importantly, one of the key findings is that there is no single effective intervention, no ‘silver bullet’. Rather, ‘bundles of interventions’ backed by ‘political and executive commitment’ are needed [27].

Evidence-based interventions can be grouped into three broad themes relating to (1) *targeted enrolment into training and appropriate education* designed to produce a competent, accessible, acceptable and ‘fit-for-purpose’ workforce; (2) addressing broader health system issues to ensure *a safe and supportive work environment*; and (3) providing *ongoing individual and family support*. We now describe the interventions or workforce retention programmes associated with each of these themes, and then highlight policy implications for government and community-controlled services.

1. Appropriate education: career pathways

Educating health professionals to work in remote areas is best considered in the context of career pathways. The strongest evidence of the impact of education on future rural or remote practice relates to the ‘integrated rural medical pipeline’, which has been developed to ameliorate the geographical maldistribution of doctors. Although little of the existing substantive literature relating to nursing workforce turnover and retention focuses specifically on the remote nursing workforce [25, 28], the broader nursing literature and some specific rural and remote health evidence describe a similar range of strategies to those targeting the components of the medical ‘pipeline’ [29, 30].

Each component of the ‘pipeline’ independently contributes to increased rural practice and retention of doctors in non-metropolitan areas. The components include prioritising applicants with rural or remote backgrounds [31–35], and prioritising Indigenous student entry, including appropriate Indigenous entry pathways [36]. Rural background or interest in rural practice and rural-focused curricula are also effective strategies in nursing [29].

Secondly, undertaking initial medical education in remote and rural areas is effective [33, 37], including a gradient effect—the longer the better [32, 38–40]. There is some evidence of a similar effect with nurses [41, 42].

Vocational and post-graduate training in rural areas is additive to rural origin [43–52], and providing financial, pedagogical and pastoral support is important, especially for Indigenous students [36, 53]. Reduction of professional and social isolation through education, adequate personal and professional support [29, 30], including preceptorship for new graduates [54], and mentoring for more experienced nurses also have an impact on nurse retention [55].

The integrated rural medical pathway can be adapted to other remote health professionals, with due regard to specific strategies targeting Indigenous health professional development, such as external study in their own communities [56]. Available evidence suggests ‘joining up’ any existing initiatives to ensure an integrated remote health workforce pathway tailored to the specific needs of all remote health workers, such that the generic pathway consists of:

- Prioritising remote origin and Indigenous students for university entry (including bridging courses for those students who have had limited educational opportunity);
- Maximising early exposure and training in remote areas;
- Contextualising the curriculum for Indigenous health and remote practice;
- Providing financial, pedagogical and pastoral support (especially for Indigenous students); and
- Ensuring a clear, supported career pathway including post-graduate and continuing professional development support, with flexible entry and exit points.

Developing a competent, ‘fit for purpose’ and stable remote health workforce requires strong intersectoral collaboration between the health and education sectors to ensure health service needs are met; together with an employment policy which prioritises Indigenous employment in remote areas with a significant Indigenous population.

2. A safe and supportive work environment: a whole of system approach

An appropriately trained and retained workforce is one component of a range of interconnected health system factors which are required to ensure a sustainable and effective remote or rural PHC service [57, 58].

First, adequate service funding is essential. We estimated an average per capita cost to NTG of remote PHC services in 2015 of A$3004, which was only 21% higher than the national average PHC expenditure figure of A$2484 [3]. Given factors of extreme isolation, transport costs and excessive disease burden in remote communities, the actual cost of meeting these needs is considerably higher. One consequence of underfunding is the inability to maintain adequate staffing levels so as to prevent work stress, burnout and staff turnover [10].

Secondly, adequate infrastructure is vital to facilitate high-quality, professionally satisfying care. This includes fit-for-purpose clinics, housing for resident and visiting staff, transport, and information and communications technology.

Thirdly, different and flexible workforce models (such as month on/month off; job-sharing; the Central Australian nurse management (CAN) model which offered a transition from acute care to remote nursing; and higher utilisation of Nurse Practitioners in remote areas) or other methods of providing respite and ongoing up-skilling of remote staff should be considered, trialled and evaluated [59]. Integral to these models and optimal retention is good ‘fit’ between individual staff and the community in which they work, including the knowledge and skills required to work in a culturally safe fashion [22, 60].

Lastly, effective co-ordination and management of services, and community participation are fundamental PHC service requirements. Management needs to be strengthened through the employment or development of qualified, competent managers who empower remote teams and create a positive practice environment [60–62]. In relation to governance, there is growing evidence that structural community participation—as evidenced by Aboriginal and Torres Strait Islander Community Controlled Health Services—results in culturally safe services that reflect the priorities of the community, improve access and health outcomes, and employ greater numbers of Indigenous people [22, 63, 64]. Recruiting and retaining local Indigenous non-clinical staff—community workers, administrative staff etc.—contributes to increasing overall stability, access and continuity of care given the need for visiting services [22, 65].

In short, the development of a competent remote health workforce within an appropriately resourced, well-managed health system that is flexible and responsive to community and staff needs is an effective retention strategy. Supportive policies include the development and implementation of an equitable, needs-based formula for funding remote health services; and policies that facilitate transition to community control, prioritise Indigenous employment and mandate a culturally safe work context.

3. Ongoing individual and family support

Hogenbirk and colleagues suggest that there is some diminution of the effect of rural origin on practice location over time, perhaps due to a lack of training opportunities or practice support in rural areas [66] As professional and personal needs change over time, other interventions are needed to support the remote practitioner, such as ensuring that financial incentives, including salary loadings or retention bonuses, are commensurate with the job. However, these are insufficient on their own and need to be part of a customised bundle of incentives that might include a retention bonus, continuing professional development opportunities, ‘time out’, psychological support and/or family support, such as educational cost support for children [12, 23, 67].

The underpinning policy relates to flexibility of employment conditions in order to be able to offer customised retention packages on a case by case basis.

## Conclusions

Optimal remote health workforce stability and preventing excessive ‘avoidable’ turnover mandate alignment of government and health authority policies with both health service requirements and individual health professional and community needs. Supportive policies which we have highlighted include:

- Strong intersectoral collaboration between the health and education sectors to ensure a fit-for-purpose workforce;
- A funding policy which mandates the development and implementation of an equitable, needs-based formula for funding remote health services;
- Policies that facilitate transition to community control, prioritise Indigenous training and employment, and mandate a culturally safe work context; and
- An employment policy which provides flexibility of employment conditions in order to be able to offer individually customised retention packages

No one intervention by itself will ensure improved retention. A ‘bundle of interventions’ is needed, drawing on the three main pillars that underpin the remote health workforce strategy outlined above, namely the development of integrated training and career pathways for all remote health professionals; ensuring a safe and supportive work environment; and meeting individual and family needs.

There are some knowledge gaps that should inform future research in this area. These include achieving a better understanding of the drivers of very high turnover in the remote health workforce and why retention is better in some communities than others [68]. Secondly, we need to implement studies which assess the association between workforce stability/retention and patient continuity of care and health outcomes. Lastly, there is a distinct lack of rigorous evaluation of retention interventions in the international literature. Any serious effort requires rigorous evaluation so that effectiveness can be monitored and programmes modified to ensure optimal impact and value for money.

Despite these gaps in knowledge, there is sufficient evidence from around the world about effective retention strategies that contribute to slowing excessive remote health workforce turnover, resulting in significant cost savings and improved continuity of care. The fundamental problem comprises an ‘implementation gap’ in translating extant empirical research evidence into actions designed to resolve existing problems. If we wish to ameliorate the very high turnover of staff in remote areas, in order to provide an adequate and equitable service to populations with arguably the highest health needs in the country, we need political and executive commitment to get the policy settings right and ensure the coordinated implementation of multiple strategies, including better linking existing strategies and ‘filling the gaps’ where necessary.

## Data Availability

Not applicable

## Abbreviations

AHP: Aboriginal health practitioner
AIHW: Australian Institute of Health and Welfare
DOH: Department of Health
FTE: Full-time equivalents
NT: Northern Territory
NTG: NT Government
p.a.: Per annum
PHC: Primary health care
RAN: Remote area nurse

## References

[1] Macinko J, Starfield B, Shi L (2003). The contribution of primary care systems to health outcomes within Organization for Economic Cooperation and Development (OECD) countries, 1970-1998. Health Services Research..

[2] Zhao Y, Thomas SL, Guthridge SL, Wakerman J (2014). Better health outcomes at lower costs: the benefits of primary care utilisation for chronic disease management in remote Indigenous communities in Australia's Northern Territory. BMC Health Serv Res..

[3] Australian Institute of Health and Welfare 2018. Australia’s Health. (Australia’s health series no. 16. AUS 221: 259-269). Canberra: AIHW;2018.

[4] Anderson Ian, Robson Bridget, Connolly Michele, Al-Yaman Fadwa, Bjertness Espen, King Alexandra, Tynan Michael, Madden Richard, Bang Abhay, Coimbra Carlos E A, Pesantes Maria Amalia, Amigo Hugo, Andronov Sergei, Armien Blas, Obando Daniel Ayala, Axelsson Per, Bhatti Zaid Shakoor, Bhutta Zulfiqar Ahmed, Bjerregaard Peter, Bjertness Marius B, Briceno-Leon Roberto, Broderstad Ann Ragnhild, Bustos Patricia, Chongsuvivatwong Virasakdi, Chu Jiayou, Deji, Gouda Jitendra, Harikumar Rachakulla, Htay Thein Thein, Htet Aung Soe, Izugbara Chimaraoke, Kamaka Martina, King Malcolm, Kodavanti Mallikharjuna Rao, Lara Macarena, Laxmaiah Avula, Lema Claudia, Taborda Ana María León, Liabsuetrakul Tippawan, Lobanov Andrey, Melhus Marita, Meshram Indrapal, Miranda J Jaime, Mu Thet Thet, Nagalla Balkrishna, Nimmathota Arlappa, Popov Andrey Ivanovich, Poveda Ana María Peñuela, Ram Faujdar, Reich Hannah, Santos Ricardo V, Sein Aye Aye, Shekhar Chander, Sherpa Lhamo Y, Skold Peter, Tano Sofia, Tanywe Asahngwa, Ugwu Chidi, Ugwu Fabian, Vapattanawong Patama, Wan Xia, Welch James R, Yang Gonghuan, Yang Zhaoqing, Yap Leslie (2016). Indigenous and tribal peoples' health (The Lancet–Lowitja Institute Global Collaboration): a population study. The Lancet.

[5] Wakerman J, Bourke L, Humphreys JS, Taylor J 2017 Is remote health different to rural health? Rural Remote Health. 17(2): 3832. doi: 10.22605/RRH3832. 10.22605/RRH3832 28549382

[6] Mason J (2013). Review of Australian Government Health Workforce Programs.

[7] Studdert L (2010). Remote Area Health Corps: nurses making a contribution to primary health services in the NT. Australas Emerg Nurs J..

[8] Busbridge MB, Smith A (2015). Fly in/fly out health workers: a barrier to quality in health care. Rural Remote Health..

[9] Hunter E. “In Indigenous health the relentless ‘pursuit of efficiency’ masks an alarming drop in care.” Weekend Australian. 2015; November 28-29.

[10] Lenthall S, Wakerman J, Opie T, Dunn S, MacLeod M, Dollard M (2011). Nursing workforce in very remote Australia, characteristics and key issues. Aust J Rural Health..

[11] Duffield CM, Roche MA, Homer C, Buchan J, Dimitrells S (2014). A comparative review of nurse turnover rates and costs across countries. J Adv Nurs..

[12] World Health Organisation. Increasing access to health workers in remote and rural areas through improved retention: Global policy recommendations. WHO: Geneva;2010. Available from: http://www.who.int/hrh/retention/guidelines/en/index.html 23741785

[13] Cherba Maria, Healey Akearok Gwen K., MacDonald W. Alexander (2019). Addressing provider turnover to improve health outcomes in Nunavut. Canadian Medical Association Journal.

[14] Northern Territory Government Department of Health. Remote area nurse safety: on-call after hours security. Darwin: NT Department of Health;2016.

[15] Wakerman J, Curry R, McEldowney R (2012). Fly in/fly out health services: the panacea or the problem? (editorial). Rural Remote Health..

[16] Wakerman J, Humphreys J, Bourke L, Dunbar T, Jones M, Carey TA et al. Assessing the Impact and Cost of Short-Term Health Workforce in Remote Indigenous Communities in Australia: A Mixed Methods Study Protocol JMIR Res Protoc. 2016 (Oct 3); 5(4): e135 Available from: http://www.researchprotocols.org/2016/4/e135 10.2196/resprot.5831 PMC5067357 27697750

[17] Russell DJ, Zhao Y, Guthridge S, Ramjan M, Jones MP, Humphreys JS et al. Patterns of resident health workforce turnover and retention in remote communities of the Northern Territory of Australia, 2013–2015. Hum Resour Health. 2017;15:52 Available from: 10.1186/s12960-017-0229-9 10.1186/s12960-017-0229-9 PMC5558760 28810919

[18] Zhao Y, Russell DJ, Guthridge S, Ramjan M, Jones MP, Humphreys JS et al. Long-term trends in supply and sustainability of the health workforce in remote Aboriginal communities in the Northern Territory of Australia. BMC Health Serv Res. 2017;19;17(1):836. Available from: doi: 10.1186/s12913-017-2803-1. 10.1186/s12913-017-2803-1 PMC5738145 29258521

[19] Department of Families Housing Community Services and Indigenous Affairs: Northern Territory Emergency Response. Evaluation Report 2011. Canberra: FaHCSIA;2011. Available from: http://hdl.handle.net/10070/252540

[20] Zhao Yuejen, Russell Deborah J., Guthridge Steven, Ramjan Mark, Jones Michael P., Humphreys John S., Wakerman John (2019). Cost impact of high staff turnover on primary care in remote Australia. Australian Health Review.

[21] Zhao Yuejen, Russell Deborah Jane, Guthridge Steven, Ramjan Mark, Jones Michael P, Humphreys John S, Wakerman John (2019). Costs and effects of higher turnover of nurses and Aboriginal health practitioners and higher use of short-term nurses in remote Australian primary care services: an observational cohort study. BMJ Open.

[22] Dunbar T, Bourke L, Murakami-Gold L. Remote Area Nurses in NT Government Clinics: More than just numbers! Aust J Rural Health. 2019;00:1–6. Available from: 10.1111/ajr.12513 10.1111/ajr.12513 31062896

[23] Buykx P, Humphreys J, Wakerman J, Pashen D (2010). Systematic review of effective retention incentives for health workers in rural and remote areas: Towards evidence-based policy. Aust J Rural Health..

[24] Russell Deborah Jane, Wakerman John, Humphreys John Stirling (2013). What is a reasonable length of employment for health workers in Australian rural and remote primary healthcare services?. Australian Health Review.

[25] Garnett S, Coe K, Golebiowska K, Walsh H, Zander K, Guthridge S, Malyon R (2008). Attracting and keeping nursing professionals in an environment of chronic labour short-age: A study of mobility among nurses and midwives in the Northern Territory of Australia.

[26] Guthrie B, Saultz JW, Freeman GK, Haggerty JL (2008). Continuity of care matters. BMJ..

[27] Kroezen M, Dussault G, Craveiro I, Dieleman M, Jansen C, Buchan J (2015). Recruitment and retention of health professionals across Europe: A literature review and multiple case study research. Health Policy..

[28] Buchan J, Couper ID, Tangcharoensathien V, Thepannya K, Jaskiewicz W, Perfilieva G (2013). Early implementation of WHO recommendations for the retention of health workers in remote and rural areas. Bull World Health Organ..

[29] Dieleman M, Kane S, Zwanikken P, GerretSen B (2011). Realist review and synthesis of retention studies for health workers in rural and remote areas.

[30] Mbemba G, Gagnon MP, Pare G, Cote J. Interventions for supporting nurse retention in rural and remote areas: an umbrella review. Hum Resour Health. 2013;11:44. Available from: 10.1186/1478-4491-11-44 10.1186/1478-4491-11-44 PMC3847170 24025429

[31] Easterbrook M, Godwin M, Wilson R, Hodgetts G, Brown G, Pong R (1999). Rural background and clinical rural rotations during medical training: effect on practice location. CMAJ..

[32] Kondalsamy‐Chennakesavan Srinivas, Eley Diann S, Ranmuthugala Geetha, Chater Alan B, Toombs Maree R, Darshan Deepak, Nicholson Geoffrey C (2015). Determinants of rural practice: positive interaction between rural background and rural undergraduate training. Medical Journal of Australia.

[33] Kwan Marcella M. S., Kondalsamy-Chennakesavan Srinivas, Ranmuthugala Geetha, Toombs Maree R., Nicholson Geoffrey C. (2017). The rural pipeline to longer-term rural practice: General practitioners and specialists. PLOS ONE.

[34] Laven Gillian, Wilkinson David (2003). RURAL DOCTORS AND RURAL BACKGROUNDS: HOW STRONG IS THE EVIDENCE? A SYSTEMATIC REVIEW. Australian Journal of Rural Health.

[35] Rabinowitz HK, Diamond JJ, Markham FW, Hazelwood CE (1999). A program to increase the number of family physicians in rural and underserved areas: impact after 22 years. JAMA..

[36] Behrendt L, Larkin S, Griew R, Kelly P (2012). Review of Higher Education Access and Outcomes for Aboriginal and Torres Strait Islander People: Final Report.

[37] Halaas Gwen Wagstrom, Zink Therese, Finstad Deborah, Bolin Keli, Center Bruce (2008). Recruitment and Retention of Rural Physicians: Outcomes From the Rural Physician Associate Program of Minnesota. The Journal of Rural Health.

[38] Farmer J, Kenny A, McKinstry C, Huysmans RD. A scoping review of the association between rural medical education and rural practice location. Hum Resour Health. 2015;13:27. Available from: DOI 10.1186/s12960-015-0017-3 10.1186/s12960-015-0017-3 PMC4436115 25943870

[39] Playford Denese, Puddey Ian B. (2016). Interest in rural clinical school is not enough: Participation is necessary to predict an ultimate rural practice location. Australian Journal of Rural Health.

[40] Zink Therese, Center Bruce, Finstad Deborah, Boulger James G., Repesh Lillian A., Westra Ruth, Christensen Raymond, Brooks Kathleen Dwyer (2010). Efforts to Graduate More Primary Care Physicians and Physicians Who Will Practice in Rural Areas: Examining Outcomes From the University of Minnesota–Duluth and the Rural Physician Associate Program. Academic Medicine.

[41] Chenoweth L, Jeon Y, Merlyn H, Brodaty H (2010). A systematic review of what factors attract and retain nurses in aged and dementia care. J Clin Nurs..

[42] Gordon Hene T., Denton Denise (1992). The Relationship of Rural Clinical Rotations to Where Registered Nurses Practice. The Journal of Rural Health.

[43] McGrail MR, Russell DJ, Campbell DG (2016). Vocational training of general practitioners in rural locations is critical for Australia rural medical workforce. Med J Aust.

[44] Chen F, Fordyce M, Andes S, Hart LG (2010). Which medical schools produce rural physicians? A 15-year update. Acad Med..

[45] Patterson, D. G., C. Holly Andrilla, D. F. Schmitz, R. Longenecker, and D. V. Evans. Outcomes of rural-centric residency training to prepare Family Medicine Physicians for rural practice. Policy Brief #158. Seattle, WA: WWAMI Rural Health Center, University of Washington;2016.

[46] Jamieson JL, Kernahan J, Calam B, Sivertz KS (2013). One program, multiple training sites: does site of family medicine training influence professional practice location?. Rural Remote Health..

[47] Bowman RC, Penrod JD (1998). Family practice residency programs and the graduation of rural family physicians. Fam Med..

[48] Rourke JT, Incitti F, Rourke LL, Kennard M (2005). Relationship between practice location of Ontario family physicians and their rural background or amount of rural medical education experience. Can J Rural Med..

[49] Heng D, Pong RW, Chan BT, Degani N, Crichton T, Goertzen J (2007). Graduates of northern Ontario family medicine residency programs practise where they train. Can J Rural Med..

[50] Pathman DE, Steiner BD, Jones BD, Konrad TR (1999). Preparing and retaining rural physicians through medical education. Acad Med..

[51] Hogenbirk JC, Timony PE, French MG, Strasser R, Pong RW, Cervin C (2016). Milestones on the social accountability journey: Family medicine practice locations of Northern Ontario School of Medicine graduates. Can Fam Physician..

[52] Fleming PM, Sinnot ML (2018). Rural physician supply and retention: factors in the Canadian context. Can J Rural Med..

[53] Anonson JM, Desjarlais J, Nixon J, Whiteman L, Bird A (2008). Strategies to Support Recruitment and Retention of First Nations Youth in Baccalaureate Nursing Programs in Saskatchewan. Canada. J Transcult Nurs..

[54] Salt J, Cummings GG, Profetto-McGrath J (2008). Increasing retention of new graduate nurses: a systematic review of interventions by healthcare organizations. J Nurs Admin..

[55] Lartey S, Cummings G, Profetto-McGrath J (2014). Interventions that promote retention of experienced registered nurses in health care settings: a systematic review. J Nurs Manag..

[56] Curtis E, Wikaire E, Stokes K, Reid P (2012). Addressing indigenous health workforce inequities: a literature review exploring ‘best’ practice for recruitment into tertiary health programmes. Int J Equity Health..

[57] Humphreys JS, Wakerman J, Wells R, Kuipers P, Jones J, Entwistle P (2008). 'Beyond workforce': a systemic solution for health service provision in small rural and remote communities. Med J Aust..

[58] Wakerman John, Humphreys John S (2012). Sustainable workforce and sustainable health systems for rural and remote Australia. The Medical Journal of Australia.

[59] van Haaren M, Williams G (2000). Central Australian nurse management model (CAN Model): a strategic approach to the recruitment and retention of remote-area nurses. Aust J Rural Health..

[60] Tyrrell Michael S., Carey Timothy A., Wakerman John (2018). The work motivations of the health practitioner who stays for a substantial time in the very remote Indigenous community workplace. Australian Journal of Psychology.

[61] Cowden T, Cummings G, Profetto-McGrath J (2011). Leadership practices and staff nurses’ intent to stay: a systematic review. J Nurs Manag..

[62] Twigg D, McCullough K (2014). Nurse retention: A review of strategies to increase and enhance positive practice environments in clinical settings. Int J Nurs Stud..

[63] Bath Jessamy, Wakerman John (2015). Impact of community participation in primary health care: what is the evidence?. Australian Journal of Primary Health.

[64] Panaretto Kathryn S, Wenitong Mark, Button Selwyn, Ring Ian T (2014). Aboriginal community controlled health services: leading the way in primary care. Medical Journal of Australia.

[65] Pulver LJ, Fitzpatrick S, Ritchie J, Norrie M (2010). Filling the gap: an evaluation of a voluntary dental program within an Aboriginal and Torres Strait islander community controlled primary health service. Aborig Isl Health Work J..

[66] Hogenbirk John C., McGrail Matthew R., Strasser Roger, Lacarte Sara A., Kevat Ajay, Lewenberg Michael (2015). Urban washout: How strong is the rural-background effect?. Australian Journal of Rural Health.

[67] Gardiner Maria, Kearns Hugh, Tiggemann Marika (2013). Effectiveness of cognitive behavioural coaching in improving the well-being and retention of rural general practitioners. Australian Journal of Rural Health.

[68] Jones Michael, Humphreys John S., McGrail Matthew R. (2012). Why does a rural background make medical students more likely to intend to work in rural areas and how consistent is the effect? A study of the rural background effect. Australian Journal of Rural Health.

